# Population structure and genetic diversity of *Bromus tectorum* within the small grain production region of the Pacific Northwest

**DOI:** 10.1002/ece3.3386

**Published:** 2017-09-07

**Authors:** Nevin C. Lawrence, Amber L. Hauvermale, Amit Dhingra, Ian C. Burke

**Affiliations:** ^1^ Department of Crop and Soil Sciences Washington State University Pullman WA USA; ^2^ University of Nebraska‐Lincoln Scottsbluff NE USA; ^3^ Department of Horticulture Washington State University Pullman WA USA

**Keywords:** agroecosystem, genotype‐by‐sequencing, postinvasion biology, single nucleotide polymorphism

## Abstract

*Bromus tectorum* L. is an invasive winter annual grass naturalized across the United States. Numerous studies have investigated *B. tectorum* population structure and genetics in the context of *B. tectorum* as an ecological invader of natural areas and rangeland. Despite the wealth of information regarding *B. tectorum*, previous studies have not focused on, or made comparisons to, *B. tectorum* as it persists in individual agroecosystems. The objectives of this study were to assess the genetic diversity and structure, the occurrence of generalist and specialist genotypes, and the influence of climate on distribution of *B. tectorum* sourced exclusively from within small grain production regions of the Pacific Northwest. Genetic diversity of *B. tectorum* sourced from agronomic fields was found to be similar to what has been observed from other land use histories. Six distinct genetic clusters of *B. tectorum* were identified, with no evidence to indicate that any of the genetic clusters were better adapted to a particular geographical area or climate within the region. Given the apparent random spatial distribution of *B. tectorum* genetic clusters at the spatial scale of this analysis, unique genotypes may be well mixed within region, similar to what was reported for other inbreeding weedy grass species.

## 1 INTRODUCTION

Agricultural weeds represent the ecological and evolutionary response of human crop cultivation to native and introduced flora (Neve, Vila‐Aoib, & Roux, 2009). Anthropogenic impacts associated with agriculture lead to fragmentation and simplification of natural ecosystems at multiple scales. The yearly disturbance of tillage, planting, and herbicide applications may impact how evolutionary forces such as genetic drift, selection, and breeding systems act against weed species in a different way than previously observed in rangeland or natural areas (Thrall et al., 2011). Neve et al. (2009) argues modern weed management requires an approach based in evolutionary biology, of which the first step is understanding “the extent, structure, and significance of genetic variation.”

Downy brome (*Bromus tectorum*) is a widely distributed weed across North America, and the population genetics of the species has been well characterized. Despite the wealth of information regarding the genetic diversity of downy brome, previous studies have not focused on, or made comparisons of downy brome genetic structure in agronomic fields. The lack of downy brome genetic studies within agroecosystems is significant given that downy brome is a widely distributed and a serious pest in small grains and other crops across North America.

Previous studies have solely investigated downy brome population genetics in the context of downy brome as an ecological invader. Consequently, previous research has focused on the prevalence of common versus rare genotypes across the landscape, genetic differences between native populations of Eurasia and invasive populations of North America, and evidence of local adaptation to distinct ecosystems (Leger et al., 2009; Merrill, Meyer, & Coleman, 2012; Novak & Mack, 1993, 2016; Novak, Mack, & Soltis, 1991, 1993; Scott et al., 2010). Novak et al. (1991) reported that a limited number of genotypes were found distributed widely across North America. In comparing native and introduced populations, total genetic diversity across the entire native range was higher than the introduced range. However, within a population, genetic diversity was greater in the introduced range (Novak & Mack, 1993, 2016). Genetic differences between native and introduced ranges can be explained by the founder effect reducing genetic diversity in the introduced range coupled with mixed populations of selfing individuals from diverse origins (Novak & Mack, 1993, 2016).

While widely spread genotypes across the introduced range can be attributed to generalists, evidence for local adaptation to an environment by specialist genotypes has been reported for downy brome. When local adaption was observed, variation in phenological traits including flowering time, vernalization requirements, and timing of mature seed set was identified as driving adaptation (Ball, Frost, & Gitelman, 2004; Meyer, Nelson, & Carlson, 2004; Rice & Mack, 1991a,b). Ramakrishnan et al. (2006) found that ecological distance better predicted genetic distance of populations than physical distance, indicating that similar habitats select for similar genotypes from widely dispersed genotypes. Downy brome has been observed invading new habitats as both broadly adapted generalist genotypes and pre‐adapted specialist genotypes (Scott et al., 2010). When characterizing genotypes in the Intermountain West, historically invaded land has been largely occupied by generalist genotypes, while recently invaded land was dominated by distinct specialist genotypes (Merrill et al., 2012; Scott et al., 2010).

Previous studies have identified low genetic diversity within the species downy brome, with mean expected heterozygosity ranging from 0.002 to 0.336 within populations (Bartlett, Novak, & Mack, 2002; Meyer et al., 2013). While heterozygous individuals have been reported in the literature, outcrossing is exceedingly rare (Leger et al., 2009; Meyer et al., 2013; Novak & Mack, 1993; Valliant, Mack, & Novak, 2007). A common garden experiment was designed to encourage and quantify outcrossing at greater frequencies than would be expected in nature, and outcrossing was observed at 0.75% (Meyer et al., 2013).

The PNW small grain production can be divided into four unique agroecological classes: annual crop, annual crop‐fallow transition, crop‐fallow, and irrigated crop. Annual crop, annual crop‐fallow transition, and crop‐fallow classes are all dryland cropping systems with winter wheat as the principle crop, bringing the most economic value, within the rotation. The division of dryland agroecological classes is predominantly driven by total annual rainfall. Annual crop land can support a crop in each year of the rotation, annual crop‐fallow transition land can support a crop in 2 out of 3 years of a rotation, and crop‐fallow land can only support a crop in 1 of 2 years. When irrigation is available winter wheat is still grown, but rotational crops become more diverse and winter wheat is not a principle rotational crop (Huggins et al., 2012). As the amount of moisture, both through precipitation and irrigation, increases so does the intensity of the cropping systems. Within the PNW small grain production region downy brome generalist genotypes would be expected throughout the entirety of the region, while specialists would be expected at greater abundance and frequency between different agroecological zones.

A genotype‐by‐sequencing approach was used in this study to estimate population structure and determine whether the genetic state of downy brome in PNW agroecosystems is similar to previous studies where individuals were sourced from nonagronomic locations. Study objectives were as follows: (1) to assess the genetic variability of downy brome sourced exclusively from within small grain production fields, (2) determine the frequency and occurrence of generalists versus specialist genotypes, and (3) determine the influence of climate on the distribution of genotypes.

## 2 METHODS

### 2.1 Sampling of plant materials

Downy brome is distributed ubiquitously within agronomic fields of the PNW. No large landscape features, such as mountain ranges, are present that could block gene flow. Climate of the region exists across a longitudinal gradient with annual precipitation in the region ranging from <300 mm to >600 mm, with precipitation increasing from the west to east. Mean annual temperature also varies on an west to east gradient, with the western portion at 11°C and decreasing to 5°C to the east based on a 30‐year average (1971; 2000) (Huggins et al., 2012). To accommodate for studies of both population genetics and structure, and for future field studies investigating climate and phenology, a systematic random sampling design was used to efficiently maximize the geographical area represented (Strofer et al., 2007).

**Figure 1:**
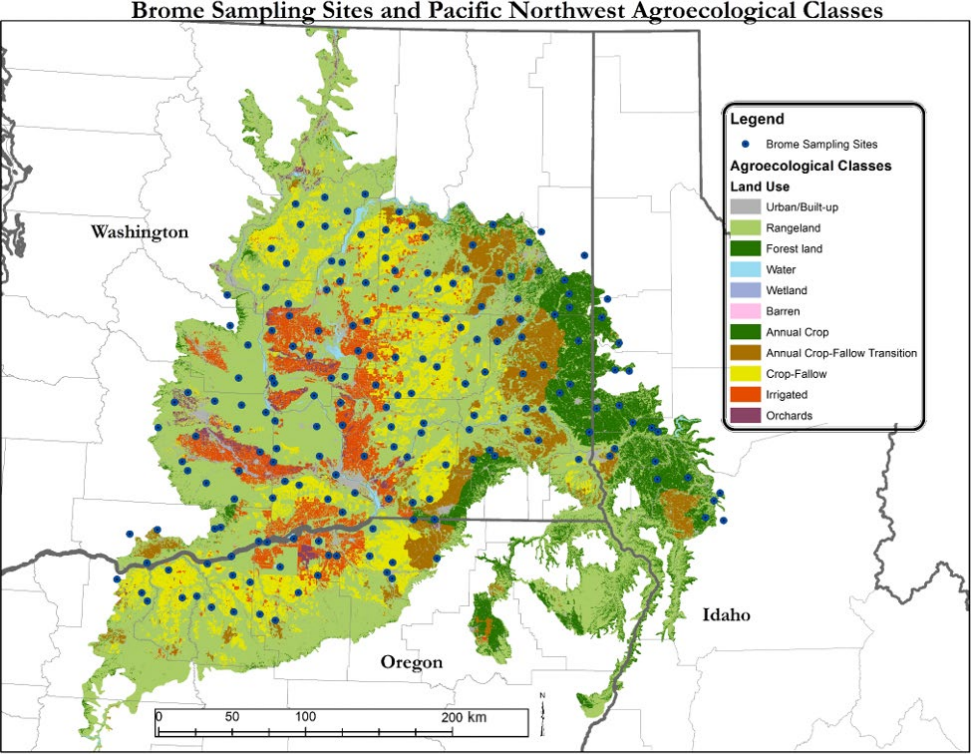
The small grain production region of the inland Pacific Northwest

A 10‐km grid was laid over the small grain production region, and a point was randomly assigned for sampling within each grid. One hundred and ninety total sampling points were generated. If the sampling point was not located in a small grain field, the sampling point was moved to the nearest small grain field. If there was no field within 3 km of the original sampling point, the location was discarded. Following re‐assignment of the original sampling locations, 130 sampling locations were retained (Figure 1).

Due to limited resources, the number of sampling locations was emphasized at the expense of collecting fewer individuals at each location (Ward & Jasieniuk, 2009). Collecting a single individual from approximately evenly spaced locations is an appropriate sampling method under the following condition: (1) the species is evenly distributed across the entire study area, (2) there are no known barriers to gene flow, (3) multilocus genetic data are used, and (4) newer Bayesian genetic clustering techniques are employed to determine genetic structure (Guillot et al., 2005, 2009; Manel et al., 2007; Strofer et al., 2007). The aforementioned criteria were satisfied given the biology of downy brome, the study location, and the methodology employed.

In June of 2010 and 2011, a single downy brome plant was collected as either mature panicles or a live plant from each of 130 re‐assigned sampling locations. Each plant was collected at least 10 m from the field border. Live plants were transplanted into a greenhouse and allowed to grow until mature panicles could be collected. Caryopses from collected panicles were later germinated to provide tissue for DNA extraction. On 21 March 2014, as plants were at the two‐ to three‐leaf stage, a single ~4‐cm leaf was collected from 95 (Table 1) of the 130 emerged downy brome collections for DNA extraction. A related species to downy brome, *Bromus diandrus* Roth (ripgut brome), was included as a control to determine whether population structure analysis could detect the related species as an outlier. DNA was extracted using a BioSprint 96 Plant Kit and BioSprint 96 workstation (Qiagen, Valencia, CA). DNA was quantified with the PicoGreen^®^ assay (Invitrogen”, Carlsbad, CA) using a Synergy” HT (BioTek^®^, Winooski, VT) microplate reader.

**Table 1:** Accession ID number, GPS coordinates of collection locations, year of collection, and cluster membership as determined by DAPC of each accession

Accession	Longitude	Latitude	Year	Agroecosystem	Accession	Longitude	Latitude	Year	Agroecosystem
1	−119.215	46.387	2010	Fallow	49	−118.37	46.677	2011	Fallow
2	−118.989	46.825	2011	Fallow	50	−119.853	46.671	2010	Fallow
3	−116.466	46.252	2010	Irrigation	51	−118.31	47.456	2011	Fallow
4	−118.916	47.785	2011	Fallow	52	−119.605	46.182	2010	Fallow
5	−120.938	45.673	2010	Fallow	53	−117.949	46.429	2010	Irrigation
6	−118.6	45.995	2011	Fallow	54	−117.748	47.903	2011	Fallow
7	−120.646	46.4	2010	Intermediate	55	−117.872	47.214	2010	Fallow
8	−118.794	46.744	2011	Annual	56	−119.935	45.388	2010	Annual
9	−118.098	46.37	2010	Fallow	57	−117.674	47.124	2010	Annual
10	−118.785	47.475	2011	Irrigation	58	−119.175	47.42	2011	Fallow
11	−120.489	45.482	2010	Irrigation	59	−117.477	46.953	2010	Fallow
12	−118.403	45.76	2010	Irrigation	60	−119.218	46.561	2010	Irrigation
13	−120.184	45.62	2010	Fallow	61^a^	−117.162	46.375	2011	Irrigation
14	−118.358	46.335	2011	Fallow	62	−119.15	47.179	2011	Fallow
15	−120.336	46.886	2011	Intermediate	63	−117.251	47.39	2010	Annual
16	−117.883	47.515	2010	Annual	64	−118.992	45.765	2011	Annual
17	−116.87	46.396	2011	Irrigation	65	−116.71	46.917	2010	Fallow
18	−118.127	46.656	2011	Irrigation	66	−119.049	46.999	2011	Irrigation
19	−119.872	47.102	2011	Irrigation	67	−115.963	46.1	2010	Fallow
20	−118.135	47.686	2011	Irrigation	68	−119.164	47.99	2011	Irrigation
21	−119.851	46.737	2011	Irrigation	69	−120.616	45.469	2010	Irrigation
22	−117.804	46.629	2010	Fallow	70	−118.61	46.082	2011	Fallow
23	−119.441	45.638	2010	Annual	71	−120.699	46.676	2010	Fallow
24	−118.464	47.49	2011	Annual	72	−118.651	46.769	2011	Annual
25	−119.864	46.263	2010	Fallow	73	−120.655	46.559	2010	Fallow
26	−117.632	47.715	2011	Irrigation	74	−118.851	47.523	2011	Irrigation
27	−119.281	45.761	2010	Irrigation	75	−120.561	46.462	2010	Fallow
28^a^	−117.378	47.263	2010	Fallow	76	−118.436	46.006	2011	Irrigation
29	−119.279	46.708	2011	Fallow	77	−120.164	46.261	2010	Fallow
30	−117.165	47.102	2010	Annual	78	−118.363	47.235	2011	Fallow
31	−119.32	47.468	2011	Annual	79	−120.412	46.996	2011	Intermediate
32	−117.092	47.484	2010	Fallow	80	−117.661	46.898	2010	Annual
33	−119.691	46.742	2010	Irrigation	81	−120.13	46.375	2010	Irrigation
34	−116.836	46.924	2010	Irrigation	82	−118.168	46.803	2011	Irrigation
35	−119.078	47.203	2011	Irrigation	83	−119.799	45.353	2010	Fallow
36	−120.965	45.483	2010	Fallow	84	−117.906	46.394	2010	Fallow
37	−118.859	46.478	2010	Intermediate	85^a^	−119.711	47.337	2011	Irrigation
38	−120.746	45.635	2010	Annual	86	−118.18	46.913	2011	Intermediate
39	−118.742	46.343	2011	Annual	87	−119.373	46.107	2010	Annual
40^b^	−120.184	46.041	2010	Fallow	88	−120.241	46.003	2010	Irrigation
41	−118.642	47.261	2011	Fallow	89	−119.411	46.849	2011	Irrigation
42	−120.358	45.419	2010	Irrigation	90	−117.518	46.492	2010	Fallow
43	−118.679	47.796	2011	Fallow	91	−119.241	46.029	2010	Fallow
44	−120.162	45.396	2010	Fallow	92	−117.551	47.524	2011	Fallow
45	−118.491	46.138	2011	Intermediate	93	−119.199	47.014	2011	Fallow
46	−119.908	46.702	2010	Annual	94	−117.245	47.307	2010	Intermediate
47	−118.465	47.49	2011	Fallow	95	−119.37	47.888	2011	Annual
48	−120.346	45.895	2010	Fallow	96	−118.895	46.669	2011	Fallow

a Accession were removed from further analysis following GBS.

b Accession is Bromus diandrus Roth.

### 2.2 Genotype‐by‐sequencing

A reduced representation genotype‐by‐sequencing (GBS) approach was employed to identify SNP molecular markers (Elshire et al., 2011). A modified GBS protocol developed by Mascher et al. (2013) for use with semiconductor sequencing platform was followed. Amplicons were sequenced on an Ion Proton” sequencer using an Ion P1” Chip (Life Technologies, Carlsbad, CA). Sequencing data were obtained in FASTQ file format, and the file size ranged from 5 to 112 MBs with an average size of 45.6 MBs. Average sequence length was 100 bp, and all sequences were trimmed to 100 bp using FASTX to provide a uniform sequence length for SNP calling.

### 2.3 SNP calling

SNP calling was conducted using Stacks (1.22, Cresko Laboratory, Eugene, OR) (Catchen et al., 2013). The Stacks program aligns identical or nearly identical sequence reads into “Stacks” across individuals, and a catalog file is written. Each locus from each individual is matched against the catalog to determine the allelic state at each locus in each individual, while filtering and discarding poor‐quality reads. As there is no reference genome available for *Bromus tectorum,* the Perl script denovo_map.pl was used to call SNPs using default settings (Catchen et al., 2013). Called SNPs were filtered using the populations command in Stacks. Default parameters were used, with the exception of requiring a minimum stack depth of 5, and all loci to be found in 75% of individuals to ensure the validity of obtained markers.

### 2.4 Analysis of population structure and genetic diversity

The output from stacks was analyzed in R (R Development Core Team^2014^) using the package adegenet (Jombart, 2008; Jombart & Ahmed, 2011). Using the adegenet package, discriminant analysis of principal components (DAPC) (Jombart, Devillard, & Balloux, 2010) was used to describe population structure of collected downy brome accessions. DAPC consists of two general steps. Principal component analysis (PCA) is first used to find the optimal number of clusters (*k*), based on genetic similarity and upon Bayesian information criterion (BIC), and to initially assign individuals to each cluster. In the second step, synthetic variables called linear discriminants, consisting of linear combinations of alleles, are used to discriminate the cluster membership of each individual. SNPs which are retained by the DAPC, due to their value in discriminating cluster membership of individual accessions, can be considered “more informative SNPs” and will be referred to as such throughout the manuscript.

To complement cluster assignments based upon DAPC, the fixation index (*F*_ST_) between each genetic cluster was calculated (Nei, 1973) along with genetic distance using Nei's standard (Nei, 1972, 1978) using the R package “adegenet.” A dendrogram was then constructed from the resulting genetic distance matrix using the R package “ape” (Paradis, Claude, & Strimmer, 2004; Popescu, Huber, & Paradis, 2012). These further analyses were conducted as they retain the full complement of filtered SNPs, in contrast to the DAPC analysis which only retains a subset of the available genetic markers.

### 2.5 Population genetic metrics

In order to make comparisons with previous studies of *Bromus tectorum* genetics, and the genetics of the weed grass species, *Bromus sterilis* L and *Setaria sp*., observed and expected heterozygosity, genetic diversity, and genetic partitioning (*G*_ST_ and *G*’_ST_) were calculated across clusters using the R Package mmod (Bartlett et al., 2002; Godt & Hamrick, 1998; Green et al., 2001; Novak & Mack, 1993; Novak et al., 1991; Valliant et al., 2007; Wang, Wendell, & Dekker, 1995a,b; Winter, 2012).

Population genetic metrics were also calculated for downy brome accessions based upon the agroecological class that the samples were taken from. Given the geographical separation between sampling locations, it is unlikely that recent gene flow occurred between any of the accession. As such, any accessions grouped together for the purpose of calculating population genetic metrics cannot be considered true populations. However, calculating heterozygosity, genetic diversity, and genetic partitioning based upon cluster assignment, and the land use of the sampling locations, may aid in the detection of specialist or generalist genotypes across the landscape and compliment the DAPC analysis.

## 3 RESULTS

### 3.1 Reduced representation sequencing

Raw reads per accession ranged from 51,740 to 1,030,188 (Table 2). After trimming and filtering retained reads ranged from 741 to 13,985, per accession, from which 16,382 SNPs were initially called. SNPs that were then found in at least 75% of individuals were retained for further analysis, resulting in 384 SNPs being selected. The number of retained reads and SNPs was not uniformly retained among accessions (Table 2). The DAPC approaches employed only a subset of genetic markers which were retained for further analysis. The retained SNPs for the DAPC analysis were well distributed across all accessions. Calculated population genetic metrics utilized all SNPs including those which were not well represented across all accessions. If poor SNP representation across all accessions may bias the analyses, then it would likely be detected by disagreements between the multivariate and other employed analyses.

**Table 2:** Accession ID number, raw reads before filtering, retained reads, total SNPs called from retained reads, SNPs remaining after filtering for polymorphisms present in at least 75% of individuals, and cluster assignment

Acc	Raw reads	Retained reads	Total SNPs	Filtered SNPs	Cluster	Acc	Raw reads	Retained reads	Total SNPs	Filtered SNPs	Cluster
1	311,630	8,074	659	306	3	49	235,449	5,908	471	266	4
2	491,261	12,203	1,256	316	4	50	87,350	1,404	128	109	7
3	366,270	11,716	1,040	272	3	51	306,382	10,284	757	326	1
4	254,196	7,184	621	312	7	52	201,723	4,999	400	328	4
5	265,962	6,913	647	318	5	53	335,053	10,012	889	342	1
6	202,765	6,559	501	326	3	54	77,955	1,013	117	108	4
7	383,398	12,755	1,275	240	3	55	267,913	7,325	562	250	4
8	515,094	13,985	1,257	196	4	56	323,872	2,976	751	94	4
9	383,953	10,947	920	256	4	57	120,012	2,637	197	123	4
10	213,236	5,319	431	300	1	58	235,521	5,459	534	286	4
11	231,857	6,938	576	302	3	59	222,875	6,358	467	286	4
12	178,755	5,214	364	310	4	60	231,163	7,663	652	290	4
13	239,374	6,621	479	332	2	61^a^	46,569	¯	¯	¯	¯
14	157,885	4,320	376	260	4	62	330,976	9,553	514	230	7
15	437,108	13,123	1,344	336	7	63	245,397	7,543	514	230	1
16	274,703	7,643	533	328	3	64	144,011	2,306	215	118	4
17	201,318	5,472	477	334	7	65	89,231	1,499	121	93	4
18	249,234	7,282	557	342	1	66	111,615	2,168	184	126	2
19	255,478	7,388	551	342	4	67	159,917	3,596	341	274	1
20	224,248	6,494	491	320	4	68	271,135	8,287	666	326	1
21	285,603	8,568	710	326	2	69	180,719	3,762	319	161	5
22	161,500	3,501	282	278	4	70	320,809	8,639	841	288	4
23	398,914	3,849	254	244	4	71	79,085	1,076	93	69	4
24	177,992	4,873	386	282	4	72	145,681	2,695	255	138	5
25	105,832	1,925	164	114	5	73	184,404	5,349	406	290	7
26	329,257	10,028	881	282	3	74	167,620	4,181	317	274	2
27	246,356	6,123	526	310	7	75	137,565	2,845	248	246	4
28^a^	25,870	–	–	–	–	76	94,942	1,278	131	118	4
29	325,619	10,460	941	310	4	77	104,684	1,839	144	128	7
30	216,171	5,684	468	320	4	78	89,507	1,107	116	112	2
31	98,193	1,025	114	77	4	79	232,352	6,132	569	254	4
32	160,074	4,240	271	168	4	80	132,611	2,493	253	142	1
33	225,757	5,875	395	286	4	81	227,168	5,839	501	304	5
34	211,269	6,194	489	302	3	82	204,385	6,089	452	284	5
35	86,627	941	107	105	4	83	128,435	2,760	267	157	2
36	145,952	3,051	286	144	3	84	73,498	749	118	105	2
37	401,706	11,465	1,034	322	7	85^a^	31,665	–	–	–	–
38	174,952	3,774	334	302	7	86	193,443	4,682	391	260	1
39	117,666	1,855	176	113	4	87	147,353	3,353	346	280	4
40^b^	216,529	5,557	415	214	6	88	226,010	5,679	594	318	7
41	301,680	8,453	702	354	7	89	139,471	2,862	215	212	4
42	260,351	6,941	590	344	1	90	129,576	2,367	297	296	4
43	446,979	11,679	1,219	256	4	91	85,637	1,229	207	186	1
44	90,759	1,952	138	101	4	92	146,609	2,525	271	268	7
45	91,137	1,424	114	110	1	93	155,940	3,154	306	298	4
46	138,555	2,556	246	238	7	94	363,292	11,190	955	308	2
47	146,041	2,753	212	111	4	95	113,052	2,659	250	238	4
48	323,731	8,858	780	308	4	96	177,273	4,368	398	304	4

Acc, Accession ID.

a Accession were removed from further analysis following GBS due to poor quality.

b Accession is Bromus diandrus Roth.

### 3.2 Discriminant analysis of principal components

Thirty‐five principal components were retained, corresponding to roughly 85% of cumulative variance, and used to identify seven clusters as optimal based upon BIC value. Following determination of the optimal number of clusters, multiple DAPC simulations identified six principal components as ideal in assigning group membership without overfitting the model. Three linear discriminants were retained to calculate the probability of group membership, and individuals were assigned accordingly. All clusters with the exception of cluster six contained multiple individuals. Cluster 6 contains the ripgut brome individuals (Table 1), as would be expected for the outlier individual.

**Figure 2:**
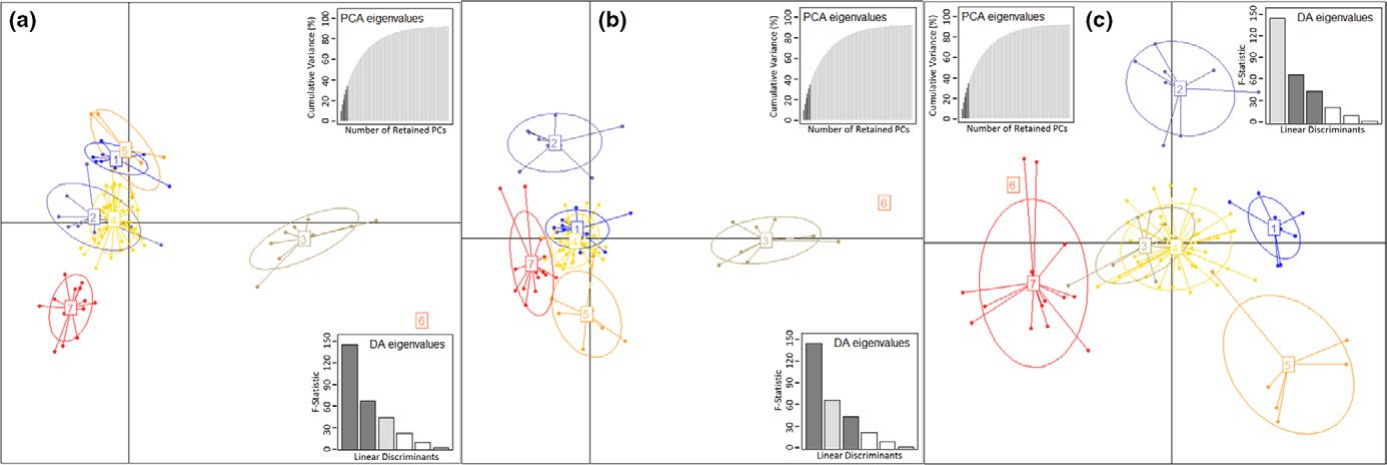
Distribution of individuals and clusters across the first, second, and third linear discriminates; PCA eigenvalues is the cumulative variance explained by the six retained principal components; DA eigenvalues represents which linear discriminants are being compared in each scatter plot, with the height of each bar representing the relative contribution in explaining total variance; scatter plot a represents linear discriminant 1, *x*‐axis, and linear discriminate 2, *y*‐axis; scatter plot b represents linear discriminant 1, *x*‐axis, and linear discriminate 3, *y*‐axis; scatter plot c represents linear discriminant 2, *x*‐axis, and linear discriminate 3, *y*‐axis; each point on each scatter plot represents an individual; each color is used to distinguish a separate cluster, which is identified by number; the ellipses around each number represent were 67% of the variance of each cluster assuming a bivariate distribution

The distribution of individuals and clusters across the first and second discriminant function (Figure 2a) indicate separation of clusters 3, 6, and 7. Cluster 2 overlapped considerably with cluster 4, as did cluster 1 with cluster 5. When individuals and clusters were distributed on the first and third discriminant function (Figure 2b) clusters 2, 5, 6 and 7 were separated, and cluster 1 overlapped with cluster 4. The distribution of clusters and individuals across the second and third discriminant function (Figure 2c) indicate overlap of the cluster 3 and 4 while clusters 1, 2, 5, 6 and 7 are distributed and distinct. Regardless of which discriminant functions were used to describe distribution, cluster 6 is the most distinct cluster. Cluster 4, however, overlaps with cluster 1, 2, and 3 depending on the linear discriminants used to describe the distribution of individuals.

*F*_ST_ values (Table 3) between each cluster reflect the differentiation between clusters described by DAPC in Figure 2. In other words, as the *F*_ST_ approaches zero there is a greater likelihood that clusters exhibit low levels of genetic differentiation and should not be considered separate from one and another. Small *F*_ST_ values were returned for cluster 4 in relation to all other clusters, 0.003–0.057, excluding cluster 6. While the sequences containing the most informative SNPs were found across all downy brome clusters, cluster 4 did not contain any of the polymorphisms of the sequences retained by DAPC. The lack of identifying SNPs for cluster 4 explains the limited dispersion of cluster 4 and low pairwise *F*_ST_ values. Cluster 6, which contained the single ripgut brome accession, was more dispersed across the linear discriminants relative to other clusters, and the dispersion indicated by DAPC was also represented by *F*_ST_ values (Table 3).

**Table 3:** Pairwise *F*_ST_ values of the 7 described genetic clusters

	Fixation index (*F*_ST_)					
	1	2	3	4	5	6
2	0.226	–	–	–	–	–
3	0.148	0.187	–	–	–	–
4	0.006	0.003	0.006	–	–	–
5	0.146	0.364	0.238	0.057	–	–
6	0.751	0.713	0.805	0.134	0.680	–
7	0.151	0.162	0.121	0.011	0.258	0.705

**Figure 3:**
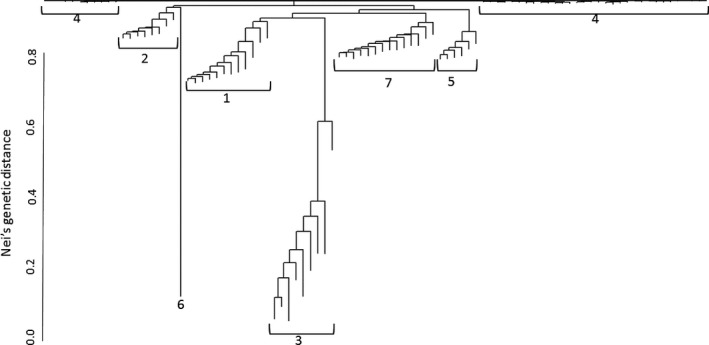
Dendrogram, calculated using Nei's genetic distance, illustrating the relatedness of the seven described genetic clusters. Each branch, running vertical, represents an accession, while horizontal bars and numbers designate cluster membership

**Figure 4:**
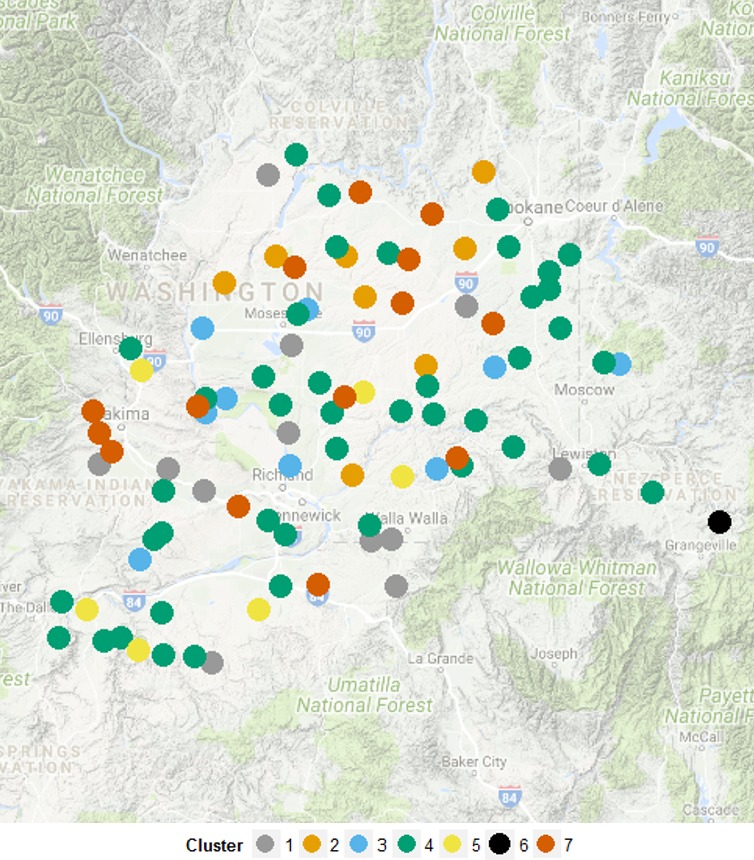
Spatial distribution of individuals and cluster membership as determined by discriminate analysis of principal component

Pairwise genetic distance values among accessions, when viewed as a dendrogram (Figure 3), resulted in a similar grouping of accessions as the DAPC analysis. Little differentiation, based upon genetic distance, is observed between clusters 1, 2, 4, 5, and 7. Cluster 6, as reflected by *F*_ST_ values, is an outlier; however, cluster 3 also appears distinct from all other clusters. The genetic distance between cluster 3 and all other clusters (Figure 3) is also illustrated in Figure 2a,b, but not Figure 2c. The spatial distribution of all individuals, color coded by assigned cluster, indicated no easy‐to‐interpret patterns of spatial distribution (Figure 4).

### 3.3 Heterozygosity, genetic diversity, and genetic partitioning

Heterozygosity was calculated for each individual loci and averaged across all accession, and across each cluster to calculate within‐cluster genetic diversity (*H*_S_), total diversity (*H*_T_), and the ratio of genetic diversity partitioned among clusters (*G*_ST_ and *G*’_ST_) using the R Package “mmod” (Hedrick, 2005; Nei, 1973; Winter, 2012). Observed heterozygosity (*H*_O_) at individual loci averaged across all accessions ranged from 0.0 to 0.65 with a mean value of 0.006. Across all accessions, expected heterozygosity at each loci (*H*_E_) ranged from 0.021 to 0.667 with a mean value of 0.2. Within‐cluster expected heterozygosity ranged from 0.069 to 0.144 with an average of 0.122 (*H*_E_) (Table 4). Within‐cluster genetic diversity was 0.085, and total genetic diversity across clusters was 0.267. Genetic partitioning was analyzed between clusters with cluster 6, the outlier cluster, removed. Partitioning of genetic diversity within and among clusters was calculated as 0.680 and 0.785, using *G*_ST_ and *G*’_ST,_ respectively, indicating that a majority of genetic diversity is partitioned among genetic clusters.

**Table 4:** Genetic diversity of *Bromus tectorum* collected from the small grain production region of the PNW

Genetic diversity by cluster			
Cluster	*H*_O_	*H*_E_
1	0.028	0.076
2	0.058	0.110
3	0.029	0.144
4	0.076	0.069
5	0.056	0.138
6^a^	0.287	0.144
7	0.025	0.101
*H*_S_	*H*_T_	*G*_ST_	*G*’_ST_
0.085	0.267	0.680	0.785

Genetic diversity by Agroecosystem			
Class	*H*_O_	*H*_E_
Annual	0.011	0.161
Transition	0.007	0.244
Fallow	0.004	0.129
Irrigated	0.005	0.249
*H*_S_	*H*_T_	*G*_ST_	*G*’_ST_
0.203	0.215	0.057	0.094

*H*_O_, observed heterozygosity; *H*_E_, expected heterozygosity; *H*_S_, within‐cluster genetic diversity; *H*_T_, total diversity; *G*_ST_ and *G*’_ST_, ratio of genetic diversity partitioned among population calculated using different mathematical formulas.

a Cluster six includes the single individual of the species *Bromus diandrus*, which was excluded in calculating *H*_S_, *H*_T_, *G*_ST_, *G*’_ST_ and from calculation of genetic diversity by Agroecosystem Class.

When heterozygosity was calculated for accessions grouped by the agroecological class, there were no substantial differences compared to when accessions were grouped by cluster. However, there was a large difference in genetic partitioning (Table 4). The majority of genetic diversity was partitioned within agroecologic classes, as indicated by *G*_ST_ and *G*’_ST_ values of 0.057 and 0.094, respectively. Comparing the results of genetic partitioning between accessions grouped by cluster and accessions grouped by agroecological classes, accessions within clusters are genetically similar, while agroecological class from where an accession was sourced has limited influence on genetic properties across the PNW.

## 4 DISCUSSION

Consistent with other studies, downy brome collected from small grain production fields in the PNW does not appear to have greater genetic diversity than populations in nonagronomic settings (Ashley & Longland, 2009; Bartlett et al., 2002; Meyer et al., 2013; Novak & Mack, 1993; Novak et al., 1991; Ramakrishnan et al., 2002; Valliant et al., 2007). Previous studies utilizing 25 allozyme markers reported observed heterozygosity ranging from 0.000 to 0.002 and expected heterozygosity ranging from 0.0 to 0.032 (Bartlett et al., 2002; Novak & Mack, 1993; Novak et al., 1991; Valliant et al., 2007). Later studies utilizing seven simple sequence repeat (SSR) markers reported greater genetic diversity compared to previous work with allozymes with observed heterozygosity ranging from 0.000 to 0.011 and expected heterozygosity ranging from 0.018 to 0.547 (Ashley & Longland, 2009; Kao, Brown, & Hufbauer, 2008; Ramakrishnan et al., 2002). Compared to the allozyme and microsatellite data, Meyer et al. (2013) reported greater observed heterozygosity, 0.001–0.009, and expected heterozygosity, 0.149–0.336, using 93 SNP markers. Within the PNW small grain production region, the average observed heterozygosity, 0.05, was greater than previous research using allozymes and SSR makers but similar to Meyer et al. (2013). Average expected heterozygosity within the PNW, 0.085, was between what was reported from allozyme and SSR marker data sets (Table 3). Higher observed heterozygosity would be expected from Meyer et al. (2013) as the sampled populations had been chosen because high rates of outcrossing were expected, based upon previous sampling which indicated relatively high heterozygosity and genetic diversity within the populations.

*G*_ST_ values from introduced *B. tectorum* populations have been previously reported as ranging from 0.241 to 0.582 (Bartlett et al., 2002; Novak & Mack, 2016; Novak et al., 1991; Valliant et al., 2007). However, within the native range of *B. tectorum* a *G*_ST_ value of 0.754 has been reported, indicating greater population differentiation within the native range of *B. tectorum* compared to the introduced range (Novak & Mack, 1993). Within the small grain production fields of the PNW, *G*_ST_ was calculated at 0.680 for accessions grouped by cluster, closer to the native range value and indicating a greater degree of population differentiation than what had previously been reported within the introduced range. Within its native range, downy brome exists as geographically isolated populations, while introduced populations typically consist of a mixture of several genotypes from unique founder events coexisting within a single location (Merrill et al., 2012; Scott et al., 2010). As this study used genotype to define clusters within a geographical region rather than defining populations by the geographical proximity of the sampling locations, greater population differentiation would be expected.

As the western PNW is considerably dryer and warmer than the eastern PNW, it was hypothesized that evidence of specialist genotypes would be found when comparing the eastern and western portions of the region. The small genetic partitioning values returned when comparing accessions by the agroecological class from which they were sourced indicates that land use class, which is predominately driven by climate, has limited influence on genetic partitioning. If strong genetic partitioning was found based upon the land class from which accessions were sourced, it would be evidence of specialist genotypes occupying specific niches based on climate or agronomic practices. Results do not suggest segregation of genotypes between the eastern and western portions of the region or by agroecological class. The lack of a strong or easy‐to‐interpret genetic cline may be an indication that climate is not a major driver of downy brome genotype distribution within the PNW. Downy brome might also be adaptable to a larger range of climates than represented within this study. Alternatively, the influence of climate might be more subtle than was detectable within this study.

The DAPC‐defined clusters describing downy brome genetic distribution were successful in identifying the ripgut brome individual. While some clusters contain greater numbers of individuals, it appears all clusters are distributed throughout the small grain production region and none of the genetic clusters can be described as specialist genotypes in relation to climatic variables or spatial distribution. Cluster distribution appears random, and a farm's location within the region is a poor indicator of what genotype(s) are likely to be found.

Compared to all other genetic clusters, cluster 3 appears to have a higher degree of genetic diversity when described by genetic distance (Figure 3) and expected heterozygosity (Table 4). Both of these measures were calculated utilizing all of the retained genetic markers after filtering SNPs for quality. When comparing the relation of cluster 3 to all other clusters described by the DAPC analysis, cluster 3 is also quite isolated from all other genetic clusters across the first linear discriminate (Figure 1a,b) but not across the second or third linear discriminant (Figure 1a–c). However, cluster 3 is not uniquely distributed across the study region, compared with other clusters (Figure 4). Therefore, although increased genetic diversity was reported, the diversity does not appear to be adaptively significant at a landscape scale.

Cluster 6, the ripgut brome outlier, is distinct from all other clusters across all linear discriminants used, and when comparing pairwise *F*_ST_ values and genetic distance. The genetic distinction between other clusters is often slight, but genetic clusters can be separated based upon SNP distribution. Efforts were made to evaluate cluster membership with a different number of retained PCs or with arbitrarily selected *k*‐values, and those efforts failed to identify ripgut brome as an outlier. The results returned by DAPC may accurately reflect the state of downy brome population structure within the small grain production region of the PNW: an assemblage of inbred individuals with little evidence of outcrossing and varying degrees of shared genetic history, and without strong evidence of adaptation to differing environmental influences.

While genetic markers linked to neutral gene regions, and SNPs in particular, are well suited to neutral evolutionary process such as genetic drift and gene flow (Helyar et al. 2011), such genetic markers are poor at detecting active evolutionary processes (Narum et al., 2013). Previous studies have demonstrated neutral markers can fail to detect local adaptation of population to habitats (Narum et al., 2010; Storz et al., 2009). As previous literature has demonstrated flowering time as adaptively significant and influenced by local climate, the genes responsible for regulating flowering pathways are a promising target to investigate potential adaptation of downy brome to climate (Ball et al., 2004; Meyer et al., 2004; Rice & Mack, 1991a,b). Future work will look at the functions associated with discovered SNPs in conjunction with known genes associated with regulating phenology.

Research into the population genetics and structure of related species to down brome and species with similar life histories provides further context into the adaptation of selfing grass species to the selection imposed by agronomic settings. Green et al. (2001) compared diversity of the inbreeding annual or biennial weed *Bromus sterilis* L. (barren brome) between farms located in the United Kingdom. Barren brome exists as an assemblage of unique but inbred biotypes within agronomic fields. Similar to what was found from sampling *B. tectorum* within PNW small grain fields, considerable spatial mixing of genotypes was found across all sampled farm fields (Scott et al., 2010). When low genetic diversity was found within a field, Green et al. (2001) attributed diversity to selection of locally adapted inbred biotypes.

Population genetics and structure have also been investigated within Poaceae genus *Setaria* which contains several inbreeding summer annual agronomic weed species with worldwide distribution. Surveying genetic diversity and structure of *Setaria viridis* (L.) Beauv (Wang et al., 1995a,b). Wang et al. (1995a) reported a separation in genotypes between northern and southern groups within North America. However, at smaller geographical scales, including at the farm and state level, geographical patterns were difficult to detect with some areas exhibiting high degrees of population differentiation while others were genetically identical. Wang et al. (1995a) concluded that diversity within a region is likely a result of the number of independent introductions, and the intensity and duration of natural selection.

Wang et al. (1995b) expanded the analysis of *Setaria* species to *S. glauca* (L.) P. Beauv. *S. geniculata* P. Beauv. and *S. faberi* Herrm. Within the introduced range of the United States, *S. geniculata* and *S. glauca* both exhibited lower genetic diversity than what was found within their native range and regional patterns of genetic partitioning, while *S. faberi* was nearly genetically identical worldwide based upon the isozyme markers used. In summary Wang et al. (1995a,b) described the observed diversity of *Setaria* species in the context of a review of genetic diversity of 499 plant species conducted by Godt and Hamrick (1998). While significantly different from “average” plant species, the low genetic diversity and high population differentiation of both *Setaria* and *Bromus* species are typical of self‐pollinating, invasive grass species (Green et al., 2001; Novak et al., 1991).

Given the apparently low genetic diversity and the similar genetic structure of *Bromus* and *Setaria* species within invaded and agricultural land, high levels of genetic diversity may not be essential for colonizing species. However, the use within this study of neutral markers may have masked genetic diversity conferring local adaption to novel environments (Narum et al., 2010, 2013; Storz et al., 2009). Future work will look to identify nonneutral genetic markers, which may better describe the influence of climate and human management on distribution and evolution.

Within the small grain production region of the PNW, *Bromus tectorum* clusters are highly differentiated and randomly dispersed, suggesting that generalists rather than specialist genotypes predominated across the region. The current structure of diversity within the PNW is likely the result of several independent introductions, constrained by natural selection (Novak et al., 1991; Wang et al., 1995a,b). Given that a limited number of genetic clusters were found within the PNW, management strategies could be developed to target differences in phenotype between clusters. Previous studies have identified differences in *Bromus tectorum* herbicide susceptibility, germination characteristics, and date of seed production (Ball et al., 2004; Hulbert, 1955; Klemmedson & Smith, 1964; Lawrence, Burke, & Yenish, 2014). If traits exhibiting variation can be linked to genotype, management strategies can be developed to target the specific populations in a given field. This targeted weed management approach has been called for in the literature (Baucom & Holt, 2009) but has yet to be realized. However, clusters are likely intermixed at smaller spatial scales than surveyed in this study, which may limit the implementation of genotype‐specific management strategies as well mixed and distinct genotypes could adapt quickly to management strategies.

## 5 CONCLUSIONS

Analysis of population genetics and genetic structure from downy brome collected within an agronomic region indicates that the heterozygous state of downy brome is similar, if not marginally greater, to what has been reported in previous literature. Downy brome exists within the PNW small grain production region as a series of generalist genotype clusters with limited evidence of spatial adaptation, similar to what was reported Novak et al. (1991) in a broad survey of downy brome across North America. Given the apparent random spatial distribution of downy brome clusters at the spatial scale of this analysis, unique genotypes may be well mixed within small grain fields, similar to what was reported for *Bromus sterilis* (Green et al., 2001).

To expand further upon the current reported findings, future efforts should include more samples of individuals from the same field to increase the spatial resolution of genetic inferences. Additionally, collection of individuals from nearby rangeland and natural areas may allow for the control of climate and the comparison of land use among accessions. Finally, phenotyping of collected individuals in common garden studies across several years or locations would provide traits to be compared across individuals and elucidate the results of DAPC clustering by correlating the separation of genotypes with phenotyping.

## CONFLICT OF INTEREST

The authors declare that they have no conflict of interest.

## DATA ACCESSIBILITY

Bioinformatic data are archived through the research funding agency website: https://data.reacchpna.org/resources/reacch-data-library/.

## AUTHOR CONTRIBUTIONS

Nevin Lawrence contributed to study design, collection of plant materials, data analysis, and writing of the manuscript. Amber Hauvermale contributed to writing the manuscript. Amit Dhingra contributed to the data analysis and writing of this manuscript. Ian Burke contributed to the study design, collection of plant materials, data analysis, and manuscript composition and was the principle investigator.
